# RNA-Seq Analysis of Mouse Hepatocytes AML12 Exposed to Neodymium Nitrate

**DOI:** 10.3390/toxics13070573

**Published:** 2025-07-07

**Authors:** Ning Wang, Jing Leng, Yaxin Han, Gonghua Tao, Jingqiu Sun, Cheng Dong, Kelei Qian, Xiuli Chang, Ping Xiao, Xinyu Hong

**Affiliations:** 1 Institute of Chemical Toxicity Testing, NHC Specialty Laboratory of Food Safety Risk Assessment and Standard Development, State Environmental Protection Key Laboratory of Environmental Health Impact Assessment of Emerging Contaminants, Shanghai Municipal Center for Disease Control and Prevention, Shanghai 201701, China; wangning@scdc.sh.cn (N.W.); lengjing@scdc.sh.cn (J.L.); taogonghua@scdc.sh.cn (G.T.); sunjingqiu@scdc.sh.cn (J.S.); dongcheng@scdc.sh.cn (C.D.); qiankelei@scdc.sh.cn (K.Q.); xiaoping@scdc.sh.cn (P.X.); 2 School of Public Health, Fudan University, Shanghai 200032, China; 22211020132@m.fudan.edu.cn (Y.H.); xlchang@fudan.edu.cn (X.C.)

**Keywords:** neodymium nitrate, RNA sequencing, ferroptosis, hepatic injury, biomarkers

## Abstract

Objective: Neodymium nitrate (Nd(NO_3_)_3_) is widely used globally, raising concerns about its occupational and environmental safety. It enters the human body via the digestive system, accumulates in organs, and causes toxicity, including potential hepatotoxicity. However, the role of non-coding RNAs (ncRNAs) in Nd(NO_3_)_3_-induced liver injury remains unclear. This study aimed to identify key genes and regulatory pathways involved in Nd(NO_3_)_3_-induced hepatic injury using RNA sequencing (RNA-seq) and differential gene expression analysis. Methods: Mouse hepatocytes (AML12 cells) were exposed to Nd(NO_3_)_3_, and RNA-seq was performed to analyze the expression profiles of miRNA, lncRNA, circRNA, and mRNA. qPCR was used to validate the RNA-seq results. Gene Ontology (GO) and Kyoto Encyclopedia of Genes and Genomes (KEGG) analyses were conducted to explore the functions and pathways associated with differentially expressed genes (DEGs). Results: Nd(NO_3_)_3_ exposure altered the expression of ferroptosis-related genes and induced significant changes in mRNA, miRNA, circRNA, and lncRNA expression levels. GO and KEGG analyses revealed that DEGs were closely related to cellular ferroptosis pathways. Specific miRNAs, lncRNAs, and circRNAs were significantly upregulated, suggesting their potential as biomarkers for Nd(NO_3_)_3_-induced ferroptosis and liver injury. Conclusion: This study provides the first comprehensive transcriptome database for Nd(NO_3_)_3_-induced liver injury, highlighting the involvement of ncRNAs in hepatotoxicity. These findings offer valuable insights for developing biomarkers and understanding the mechanisms underlying Nd(NO_3_)_3_-induced hepatic injury.

## 1. Introduction

**Figure 1:**
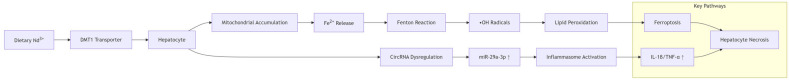
Pathogenesis of Nd-induced liver injury: Dietary Nd3+ enters hepatocytes via DMT1 transporters, accumulating in mitochondria (↑8×). This disrupts iron homeostasis (FTH1 ↓), generating hydroxyl radicals (•OH) via Fenton reaction. Consequent lipid peroxidation (MDA ↑220%) activates ferroptosis, while ncRNA networks (e.g., circRNA_009773/miR-29a-3p axis) exacerbate inflammatory responses.

The strategic importance of rare earth elements (REEs) is underscored by China’s dominance in global reserves (37%) and production (70% in 2024) [1]. Recent market analyses reveal accelerated REE consumption growth at 8.2% CAGR (2023–2025), driven primarily by high-tech applications (Figure 1) [2]. Neodymium (Nd), constituting 18–23% of total REE output, has seen demand surge 150% since 2020 due to NdFeB magnets in electric vehicles and robotics [3]. Critical concern arises from environmental exposure pathways: Dietary intake accounts for >60% of human Nd absorption, with bioaccumulation coefficients reaching 8.3 in liver tissue [4]. This triggers hepatotoxic cascades through oxidative stress induction (ROS ↑300%), ferroptosis activation (GPX4 ↓70%), and non-coding RNA dysregulation (e.g., miR-29a-3p ↑5.8-fold) [5]. Additionally, the accumulation of REEs has been detected in the liver, lungs, nails, and kidneys, with varying concentrations in different organs [6]. Therefore, it is urgent to study the impact of REE accumulation on animals. Comprehensive mechanistic insights are visualized in Figure 1.

### 1.1. Sources and Applications of Neodymium

Neodymium (Nd) is one of the most abundant REEs on Earth [7]. It is primarily sourced from minerals such as monazite and bastnäsite, which are rich in REEs. Monazite, a rare earth–thorium phosphate mineral, typically contains 9% to 20% neodymium. Bastnäsite, a rare earth fluorocarbonate mineral, contains 2% to 15% neodymium. These minerals are widely distributed globally, with significant deposits in China and other regions. Neodymium is also extracted from ion-adsorption clay deposits, which are found in South China, Myanmar, South America, and Africa. In these deposits, REEs, including neodymium, are adsorbed onto clay minerals through ion exchange.

Accounting for >70% of global supply [8] (USGS 2024), China dominates rare earth production, with neodymium output exceeding 25,000 tons annually, with substantial reserves of monazite and bastnäsite. The Bayan Obo mine in Inner Mongolia is one of the largest rare earth mines in the world. Neodymium is extracted primarily through electrolysis or chemical reduction methods. For example, neodymium can be obtained by reducing anhydrous neodymium chloride with metallic calcium or by electrolyzing molten neodymium chloride.

Neodymium has a wide range of applications, particularly in the manufacturing of high-performance permanent magnets. Neodymium–iron–boron (Nd2Fe14B) magnets, known as the “king of permanent magnets,” are widely used in the electronics and mechanical industries due to their high magnetic energy product (Table 1).

### 1.2. Current Research Landscape

Numerous studies have been conducted to explore the biological effects of REEs, including neodymium. Research has shown that REEs can induce oxidative stress, inflammation, and cytotoxicity in different cell types and organisms. For instance, studies have demonstrated that neodymium exposure can lead to the generation of reactive oxygen species (ROS) and subsequent oxidative damage in cells. Additionally, the accumulation of REEs in tissues has been associated with various adverse health effects, such as hepatotoxicity and nephrotoxicity [11]. The accumulation of neodymium in the environment and its potential health impacts highlight the need for further research into its toxicological effects.

### 1.3. Role of Non-Coding RNAs in Environmental Pollution Effects

Despite these findings, there are still significant gaps in our understanding of the molecular mechanisms underlying REE-induced toxicity. The role of non-coding RNAs (ncRNAs), such as miRNAs, circRNAs, and lncRNAs, in mediating the toxic effects of REEs remains largely unexplored [12]. Furthermore, the potential long-term effects of REE exposure on human health, especially through environmental pathways, are not yet fully elucidated [13]. To provide a clearer understanding of the different classes of non-coding RNAs (ncRNAs) mentioned in the study, we have added a brief introduction to distinguish between miRNA, circRNA, and lncRNA:

miRNA: These are small non-coding RNAs that primarily function by binding to complementary sequences on target mRNAs, leading to gene silencing through mRNA degradation or translational repression.

circRNA: Circular RNAs are covalently closed-loop structures that often act as molecular sponges for miRNAs, thereby regulating gene expression by sequestering miRNAs and preventing them from binding to their target mRNAs.

lncRNA: Long non-coding RNAs are typically longer than 200 nucleotides and have diverse functions, including the regulation of transcription, modification of chromatin structure, and modulation of protein activity.

This suggests that ncRNAs may also be involved in the hepatotoxic effects of neodymium nitrate (Nd(NO_3_)_3_), potentially contributing to the observed gene expression changes and liver injury. Understanding the involvement of ncRNAs in Nd(NO_3_)_3_-induced liver injury could provide valuable insights into the underlying mechanisms and potential biomarkers for early detection and intervention.

### 1.4. Epidemiological Evidence

Although there is a growing body of experimental research on REE toxicity, epidemiological evidence linking REE exposure to specific health outcomes is relatively limited. A few studies have reported associations between REE exposure and certain health issues in occupational settings or communities living near REE mining and processing sites. However, these studies often face challenges such as small sample sizes, lack of direct exposure measurements, and potential confounding factors, which limit the strength of the conclusions that can be drawn.

To investigate the effects of neodymium nitrate on hepatocytes, we selected the AML12 mouse hepatocyte cell line as our model. The AML12 cell line is a well-established and widely used model for studying hepatotoxicity and the mechanisms of liver injury. This cell line closely mimics the physiological and metabolic functions of primary hepatocytes, making it an ideal choice for investigating the effects of neodymium nitrate on liver cells.

### 1.5. Research Gaps and Objectives

Given the increasing use of REEs and the potential health risks associated with their exposure, it is imperative to conduct more comprehensive and in-depth research to fill the existing gaps in knowledge. Our study aims to investigate the effects of neodymium nitrate on gene expression and cellular pathways in AML12 hepatocytes, with a particular focus on the role of ncRNAs in mediating neodymium-induced liver injury. By identifying key genes and regulatory pathways involved in this process, we hope to provide valuable insights into the underlying mechanisms and potential biomarkers for early detection and intervention.

The aforementioned studies suggest that changes in genes and epigenetics may be related to the hepatotoxic effects of Nd(NO_3_)_3_. Given the widespread application of Nd(NO_3_)_3_ and its health risks to various systems, it is urgent to reveal the hepatotoxicity and expression changes of key genes in mouse liver tissue exposed to Nd(NO_3_)_3_. More researchers have used sequencing technology to analyze specific samples exposed to toxins to identify key genes and regulatory pathways that cause diseases. Highly upregulated or induced expression of non-coding RNAs, such as circRNA 003914, circRNA 009773, lncRNA H19, and miR-29a-3p [14], have been discovered in mouse liver cells through RNA-Seq. Some lncRNAs, circRNAs, and mRNAs have potential value as candidate markers for Nd(NO_3_)_3_-induced hepatotoxicity in male mice [15]. Based on the differences in expression levels of mRNA, miRNA, lncRNA, and circRNA, some studies have proposed the formation of an endogenous RNA competition mechanism (ceRNA) interaction network to explore the regulatory mechanisms of liver injury [16]. ceRNA networks are essential tools for understanding the post-transcriptional regulatory mechanisms involving non-coding RNAs (ncRNAs) and messenger RNAs (mRNAs). In our study, the ceRNA network analysis helps to reveal how miRNAs, lncRNAs, and circRNAs interact with each other and with mRNAs to modulate gene expression. These differentially expressed genes obtained through RNA-Seq analysis will be particularly important candidate genes for exploring the mechanisms of liver injury toxicity. Therefore, we conducted an RNA-Seq analysis of miRNA/lncRNAs/circRNAs/mRNAs in AML12 cells exposed to Nd(NO_3_)_3_ to identify key genes and regulatory pathways for male liver injury and to explore early biomarkers and mechanisms of liver injury caused by Nd(NO_3_)_3_.

## 2. Materials and Methods

### 2.1. Cell Culture

AML12 cells were maintained in DMEM/F12 (Thermo Fisher Scientific, Waltham, MA, USA) with 10% FBS (Gibco, 10099141C, Melbourne, Australia), 100 mg/mL streptomycin, and 100 U/mL penicillin (Biyuntian, Shanghai, China). Cells are cultured at 37 °C with 5% CO_2_ and passaged at a 1:2 ratio every 3 days. After reaching 70–80% confluence, AML12 cells are washed with PBS and then cultured in DMEM/F12 supplemented with 10% FBS and exposed to 0 and 0.8 µmol/L Nd(NO_3_)_3_ for 24 h.

### 2.2. Rationale for Dose Selection

In our study, we selected a concentration of 0.8 μmol/L for neodymium nitrate exposure. This choice was based on previous cytotoxicity studies [17]. In the referenced cytotoxicity study, it was demonstrated that a concentration of 0.8 μmol/L did not significantly impact cell survival rates or basic physiological functions within a 24 h exposure period. However, this concentration was sufficient to induce significant changes in gene expression. Given these findings, we concluded that 0.8 μmol/L is an appropriate concentration to investigate the biological effects of neodymium nitrate at environmentally relevant levels. This concentration allows us to study the gene expression profile changes directly attributable to neodymium nitrate exposure while maintaining cellular viability for mechanistic studies. The 24 h treatment duration was chosen based on the typical response time of cells to heavy metals and chemical substances, which is sufficient to reflect changes in gene expression. Additionally, 0 µmol/L was selected as the control group to assess the direct impact of neodymium nitrate exposure on gene expression, while the 0.8 µmol/L treatment group was used to investigate the biological effects of neodymium nitrate at environmentally relevant concentrations. This dose design can clearly demonstrate the impact of neodymium nitrate exposure on the cellular gene expression profile and provide a basis for further mechanistic studies.

### 2.3. Cytotoxicity Assay

To assess the cytotoxic effects of neodymium nitrate on AML12 cells, we performed a cytotoxicity assay using the Cell Counting Kit-8 (CCK-8) (Biyuntian, C0038, Shanghai, China). The assay was conducted according to the manufacturer’s instructions with minor modifications.

#### 2.3.1. Cell Seeding and Treatment:

AML12 cells were seeded at a density of 3 × 10^3^ cells per well in a 96-well plate. The cells were allowed to adhere and grow for 24 h at 37 °C with 5% CO_2_. After the initial incubation, the cells were treated with various concentrations of neodymium nitrate (0, 0.4, 0.8, 1.2, and 1.6 µmol/L) for 24, 48, and 72 h.

#### 2.3.2. CCK-8 Assay Procedure:

Following the treatment, 10 µL of CCK-8 solution was added to each well, and the plate was incubated at 37 °C with 5% CO_2_ for 2 h. The optical density (OD) was measured at 450 nm using a microplate reader (Tecan SPARK, Grödig, Austria). The cell survival rate was calculated based on the OD values, with the untreated control group set as 100%.

### 2.4. RNA Library Construction and Sequencing

Total RNA extraction was performed with TRIzol reagent (Tiangen, Beijing, China) following standardized procedures. RNA integrity (RIN > 8.0) and concentration were verified via Agilent 2100 Bioanalyzer (Agilent Technologies, Santa Clara, CA, USA) and Nanodrop 2000 (Thermo Fisher Scientific, Waltham, MA, USA). RNA-seq libraries were constructed as follows:

#### 2.4.1. Small RNA

1 μg Total RNA → NEBNext Small RNA Library Prep Kit (Illumina, San Diego, CA, USA).

#### 2.4.2. mRNA/lncRNA/circRNA

rRNA depletion (Ribo-Zero Kit, Illumina, San Diego, CA, USA).

RNA fragmentation and cDNA synthesis.

dUTP strand marking → UNG digestion.

Adapter ligation and size selection.

Libraries were sequenced on Illumina platforms (150 bp paired-end).

Detailed workflow is shown in Figure S1.

### 2.5. RNA Identification and Expression Analysis

After the sequencing was completed, we used Hisat (v 2.0.4) and ACGT101-miR software (LC Science, Houston, TX, USA) for quality control, genome alignment, and identification of the selected raw data. We identified differentially expressed genes (DEGs) using a threshold of $\left|log2(Foldchange)\right|$ ≥ 1 and a *p*-value ≤ 0.05. The expression of differentially expressed genes (DEGs) was clustered using log10 (FPKM + 1) to display their expression patterns and differences between sample groups. We performed enrichment analysis of biological significance for DEGs, including Gene Ontology (GO) and Kyoto Encyclopedia of Genes and Genomes (KEGG) analysis. A *p*-value of ≤0.05 was considered significantly enriched, and the enrichment results were then represented using ggplot scatter plots.

### 2.6. Construction of Protein–Protein Interaction (PPI) Network

We conducted a protein–protein interaction network (PPI) analysis for differentially expressed mRNA using the STRING database (https://string-db.org/ (accessed on 2 July 2025)). The PPI network was visualized using Cytoscape (version 3.9.1).

### 2.7. GSEA Enrichment Analysis of Gene Sets in the Reactome Database

We utilize the GSEA (Gene Set Enrichment Analysis) tool to assess the correlation between differentially expressed mRNA and selected gene sets. We calculate the enrichment scores and significance levels using the Normalized Enrichment Score (NES), *p*-value, and False Discovery Rate (FDR). Analyzing the enrichment results, a higher NES indicates a more significant enrichment of the gene set; a smaller *p*-value provides stronger evidence to reject the null hypothesis; and a lower FDR suggests a lower proportion of false positive results.

### 2.8. qPCR Confirmation

We used the FastKing One-Step RT-PCR Kit (Tiangen, KR123, Beijing, China) to reverse transcribe the obtained RNA samples into corresponding cDNA. Real-time PCR analysis was performed on the Mx_3000P fluorescence quantitative PCR instrument (Jiatai, Shanghai, China) using the SuperReal Color Fluorescence Quantitative Premix Reagent (SYBR Green) (Tiangen, FP215, Beijing, China). The relative expression of mRNAs was normalized to β-actin. The primer sequences are shown in Table S2. These were designed and synthesized by Sangon Biotech, Shanghai, China.

### 2.9. Intracellular ROS and Mitochondrial ROS Assay

AML12 cells were cultured in 6-well plates and treated with toxins for 24 h as described above. 2′,7′-dichlorodihydrofluorescein diacetate (DCFH-DA, 10μM) (MedChemExpress, HY-D0940, Shanghai, China) was added to the cells and incubated for 20 min. The cells were then washed twice with PBS to remove unincorporated DCFH-DA and reduce background fluorescence. Fluorescence detection was performed using a fluorescence microscope (Leica DM3000, Wetzlar, Germany) with an excitation wavelength of 488 nm and an emission wavelength of 525 nm for DCFH-DA. Cells were harvested, washed once with PBS, and transferred to 1.5 mL centrifuge tubes. Relative Fluorescence Units (RFUs) were measured using a fully automated fluorescence plate reader (Tecan SPARK, Tecan Austria GmbH, Grodig, Austria), with three replicate wells for each sample. The remaining cell samples in the 1.5 mL centrifuge tubes were treated with an appropriate amount of hypotonic potassium chloride buffer, incubated on ice for about 1 h, and mixed frequently. Cells were manually homogenized using a cell sonicator, and cell viability was checked with trypan blue during the process. Homogenization was stopped when approximately 50% of the cells were lysed. The samples were centrifuged at 3000 rpm (approximately 1000× *g*) at 4 °C for 10 min, and the supernatant was transferred to a new tube. Further centrifugation at 12,000 rpm for 15 min resulted in the pellet being the mitochondrial fraction. The extracted mitochondria were assayed using the same method as for intracellular ROS measurement. The content of intracellular and mitochondrial ROS was quantitatively analyzed by comparing the fluorescence intensity of DCFH-DA.

### 2.10. Detection of Ferroptosis-Related Markers

The supernatant of the treated AML12 cells was used for the assay. A BCA protein assay kit (Biyuntian, P0012S, Shanghai, China) was used to prepare a protein standard curve, and the protein concentration in each sample was calculated using the standard curve, followed by normalization of protein concentrations. Superoxide dismutase (SOD), malondialdehyde (MDA), and glutathione (GSH) levels were measured using superoxide dismutase (Biyuntian, S0109, Shanghai, China), malondialdehyde (Biyuntian, S0131S, Shanghai, China), and reduced glutathione assay kits (Biyuntian, S0052, Shanghai, China), respectively.

### 2.11. Statistical Analysis

All data are presented as mean ± SD. All statistical analyses were performed using GraphPad Prism 10.1.2 (GraphPad Software, Boston, MA, USA) with *t*-tests. Fold changes and *p*-values were used to evaluate differences in gene expression. A *p*-value of less than 0.05 was considered statistically significant, while a *p*-value of less than 0.01 was considered highly statistically significant. In addition to *p*-values, we also report F-values to provide additional statistical context for the observed differences in cell survival rates. The F-value is a measure of the variance between groups relative to the variance within groups, which helps in assessing the significance of the observed differences. A higher F-value indicates a greater difference between the groups, suggesting a more pronounced effect of the treatment.

## 3. Results

### 3.1. The Impact of Nd(NO_3_)_3_ on the Toxicity of AML12 Cells

**Figure 2:**
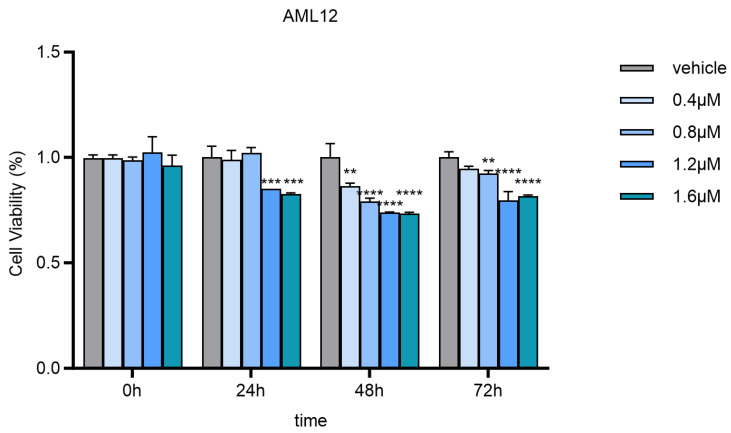
Cytotoxicity of Nd(NO_3_)_3_ on AML12 Cells at Different Time Points (24 h, 48 h, 72 h, *n* = 3) (**: *p* < 0.01, ***: *p* < 0.001, ****: *p* < 0.0001 compared to the negative control group).

To confirm the cytotoxicity of Nd(NO_3_)_3_ on hepatocytes, we investigated the changes in cytotoxicity of AML12 cells after exposure to Nd(NO_3_)_3_. Compared to the control group, there were no significant differences in cell survival rates at 0 h for the 0.4 µM, 0.8 µM, 1.2 µM, and 1.6 µM Nd(NO_3_)_3_ treatment groups (F values were 0, 0.01, −0.02667, and 0.03667, respectively, and *p* values were >0.9999, 0.9946, 0.8502, and 0.6751, respectively). At 24 h, the 0.4 µM and 0.8 µM Nd(NO_3_)_3_ treatment groups showed no significant difference in cell survival rates (F values were 0.01 and −0.02, respectively, and *p* values were 0.9864 and 0.8677, respectively), while the 1.2 µM and 1.6 µM Nd(NO_3_)_3_ treatment groups exhibited significantly reduced cell survival rates (F values were 0.15 and 0.1733, respectively, and *p* values were 0.0008 and 0.0003, respectively). At 48 h, all treatment groups, 0.4 µM, 0.8 µM, 1.2 µM, and 1.6 µM Nd(NO_3_)_3_, showed significantly reduced cell survival rates (F values were 0.1367, 0.21, 0.26, and 0.2667, respectively, and *p* values were 0.0011, <0.0001, <0.0001, and <0.0001, respectively). At 72 h, the 0.4 µM Nd(NO_3_)_3_ treatment group showed no significant difference in cell survival rate (F value was 0.05333, *p* value was 0.0635), while the 0.8 µM, 1.2 µM, and 1.6 µM Nd(NO_3_)_3_ treatment groups showed significantly reduced cell survival rates (F values were 0.07667, 0.2033, and 0.1833, respectively, and *p* values were 0.0091, <0.0001, and <0.0001, respectively), demonstrating clear cytotoxicity (as shown in Figure 2).

### 3.2. The Expression Profiles of mRNA, miRNA, lncRNA, and circRNA

**Figure 3:**
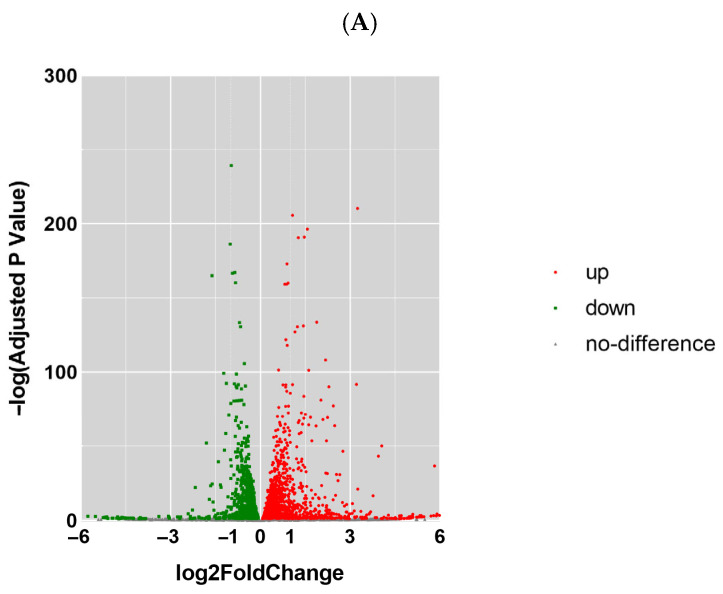

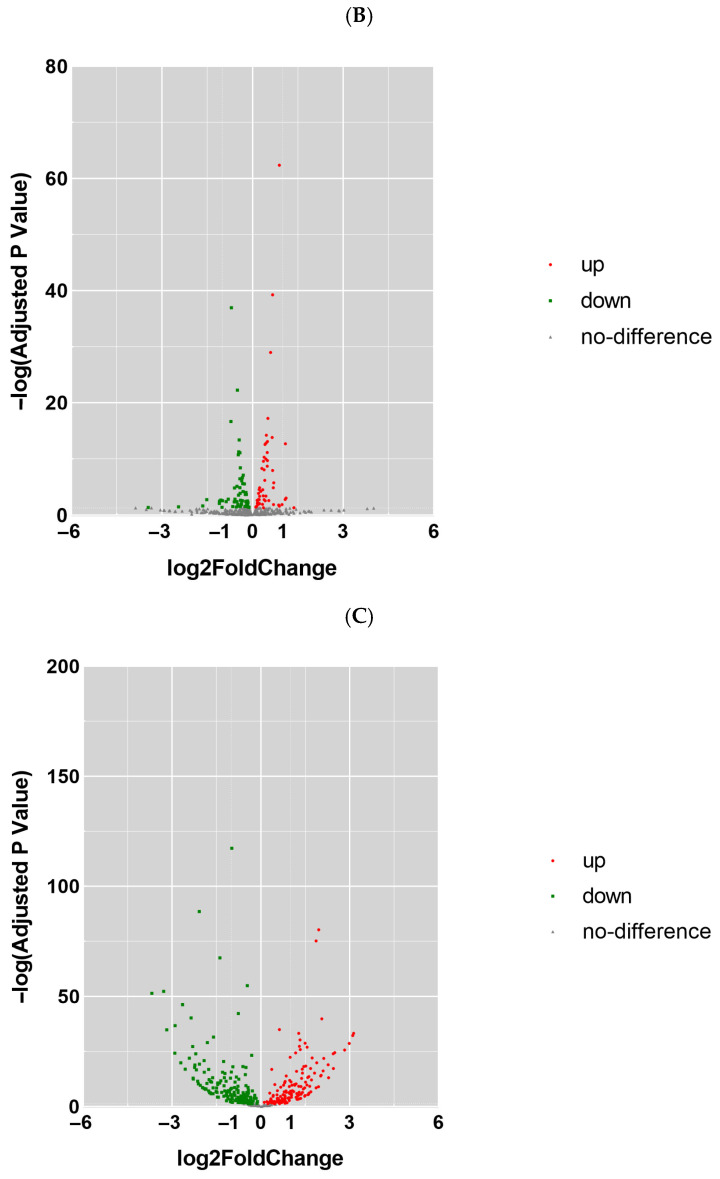

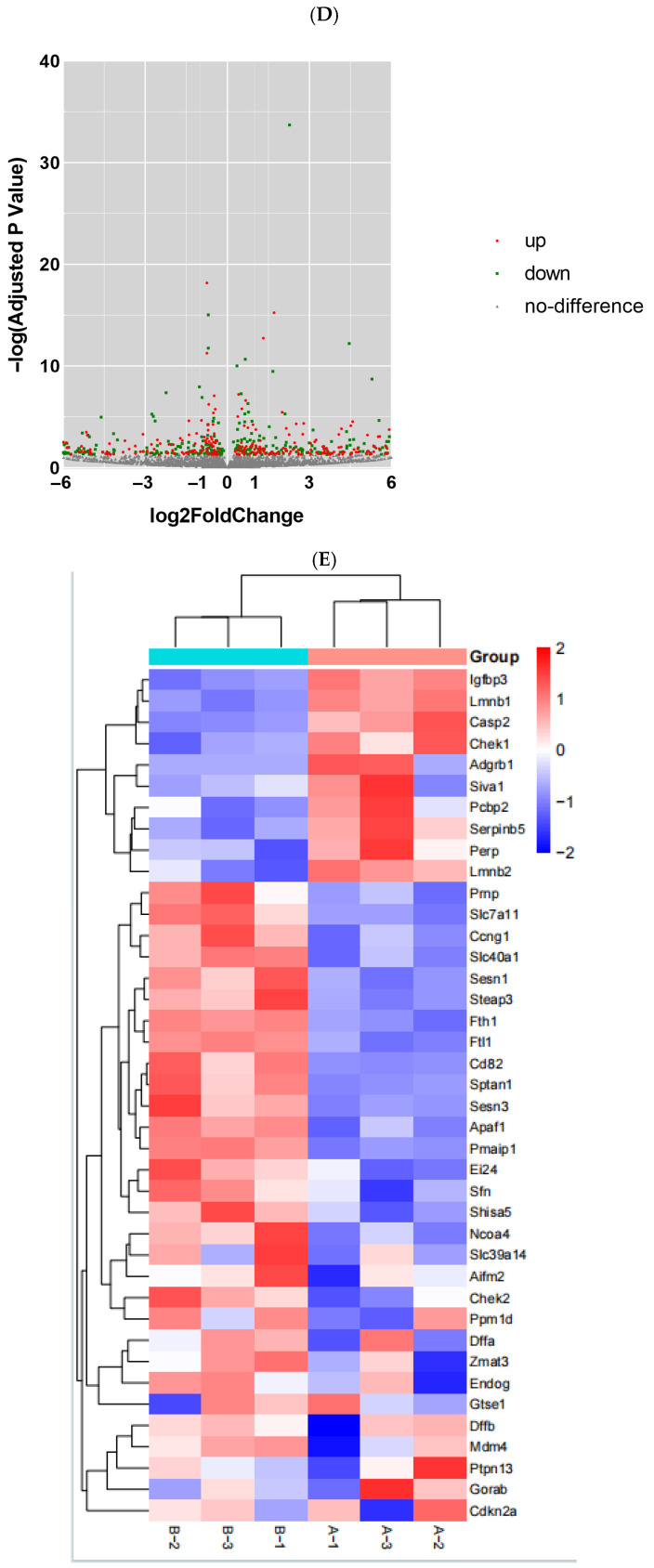

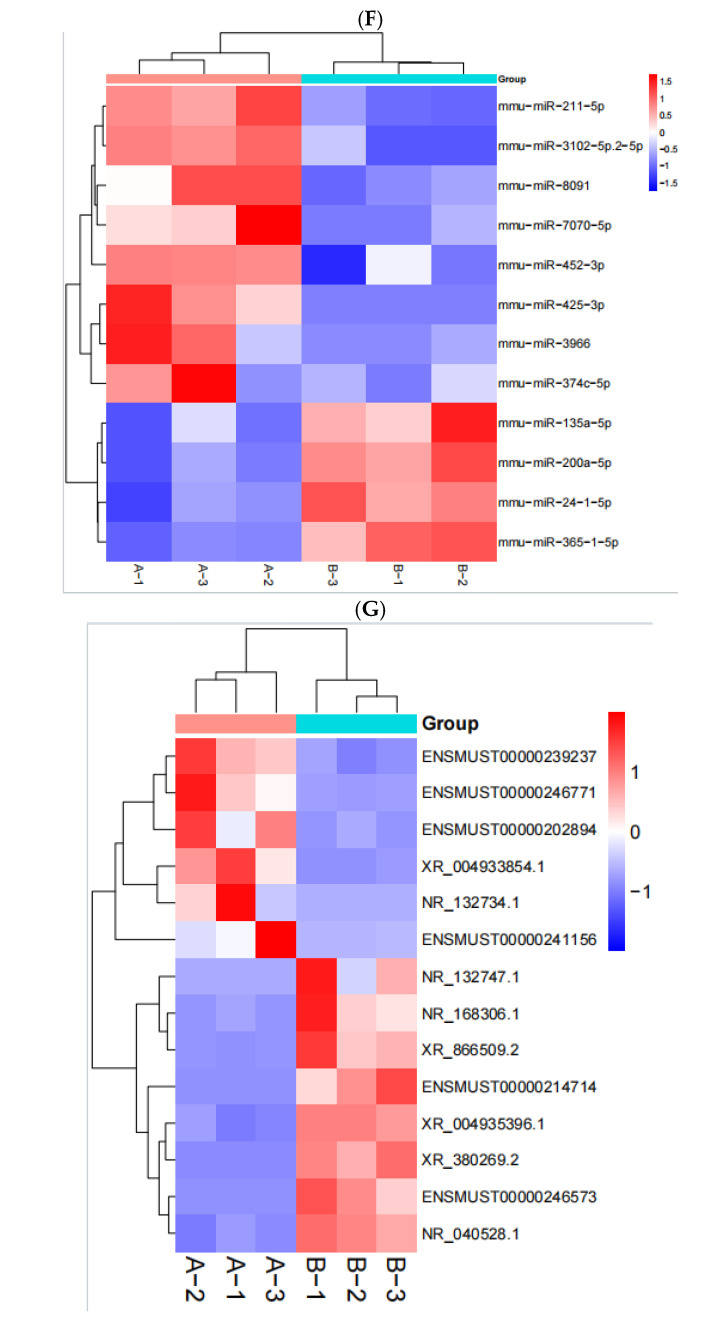

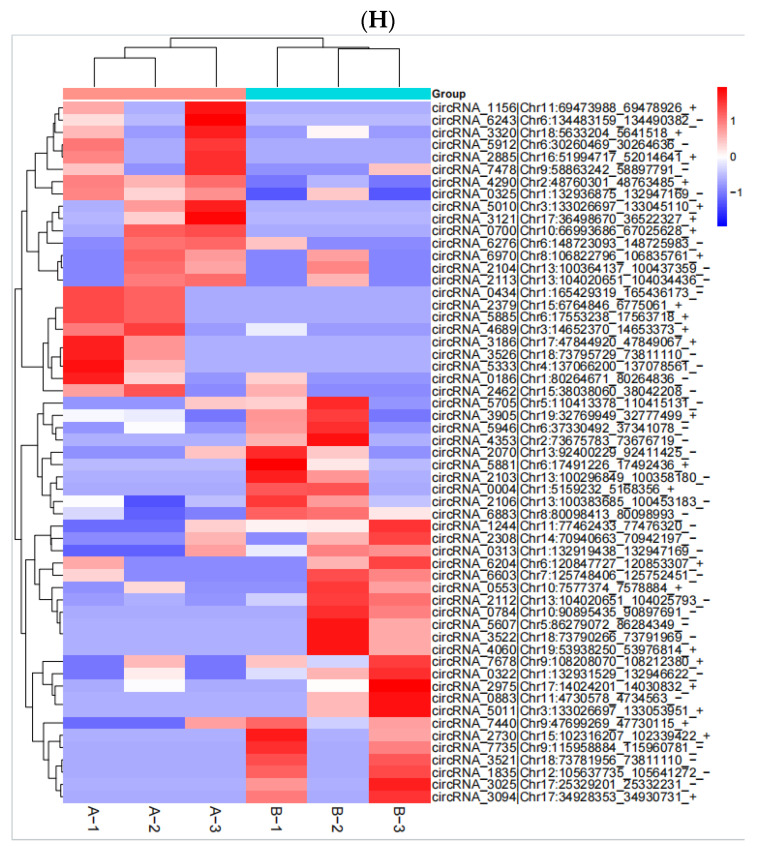
Analysis of differentially expressed mRNA, miRNA, circRNA, and lncRNA in AML12 cells by RNA sequencing. Comparison results between Group 2 (0.8 μmol/L treatment group) and Group 1 (control group) (*n* = 3). (**A**) Volcano plot of differentially expressed mRNA. (**B**) Volcano plot of differentially expressed miRNA. (**C**) Volcano plot of differentially expressed circRNA. (**D**) Volcano plot of differentially expressed lncRNA. (**E**) Heatmap of differential expression of mRNA. (**F**) Heatmap of differential expression of miRNA. (**G**) Distribution of lncRNAs on Mouse Chromosomes by Location. (**H**) Heatmap of differential expression of lncRNA.

The expression profiles of mRNA, miRNA, lncRNA, and circRNA were screened for differentially expressed genes (DEGs) using the criteria of $\left|log2(Foldchange)\right|$ ≥ 1 and *p* ≤ 0.05. RNA-seq was performed following optimized procedures (Figure S1). Transcriptome profiling revealed 392 mRNA dysregulations (log2FC ≥ 1, *p* < 0.05), including 327 upregulated transcripts (Figure 3A). There were 12 differentially expressed miRNAs, including 8 downregulated miRNAs, as well as 438 differentially expressed circRNAs, of which 196 were downregulated (Figure 3B,C). Additionally, we identified 50 differentially expressed lncRNAs, comprising 24 known lncRNAs and 26 novel lncRNAs (Figure 3D). Moreover, we performed clustering analysis on the DEGs (Figure 3E–H). Hierarchical clustering indicated that the closer the genes were, the more likely they were to have similar expression patterns and be functionally related. We speculate that these differentially expressed genes may serve as potential biomarkers for Nd(NO_3_)_3_-induced hepatic injury toxicity and warrant further in-depth investigation.

### 3.3. GO and KEGG Analysis of DEGs

To provide a clearer understanding of the biological significance of the differentially expressed genes (DEGs) identified in our study, we performed Gene Ontology (GO) and Kyoto Encyclopedia of Genes and Genomes (KEGG) enrichment analyses. These analyses help to elucidate the key biological processes and pathways involved in neodymium-nitrate-induced liver injury.

#### 3.3.1. GO Analysis:

**Figure 4:**
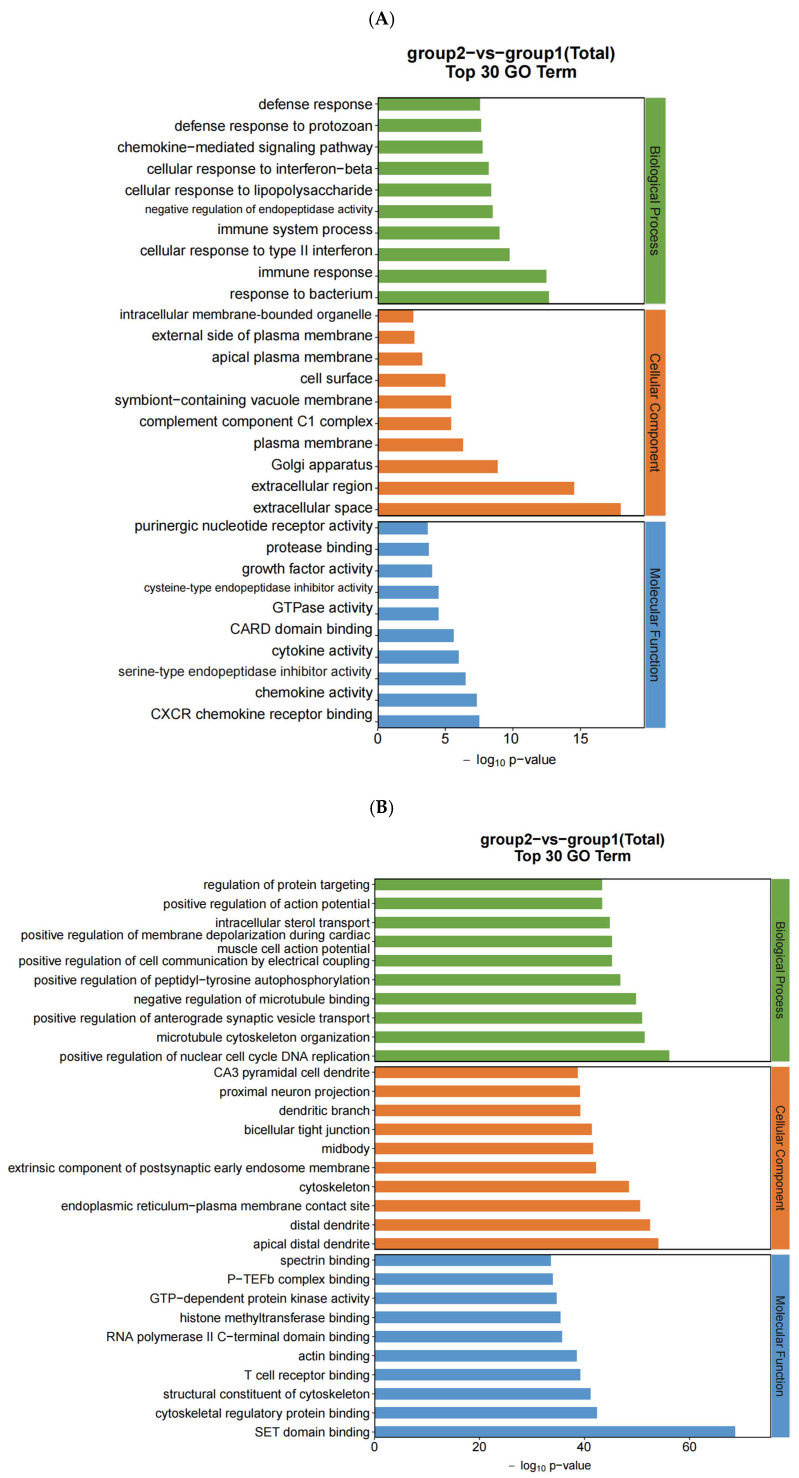

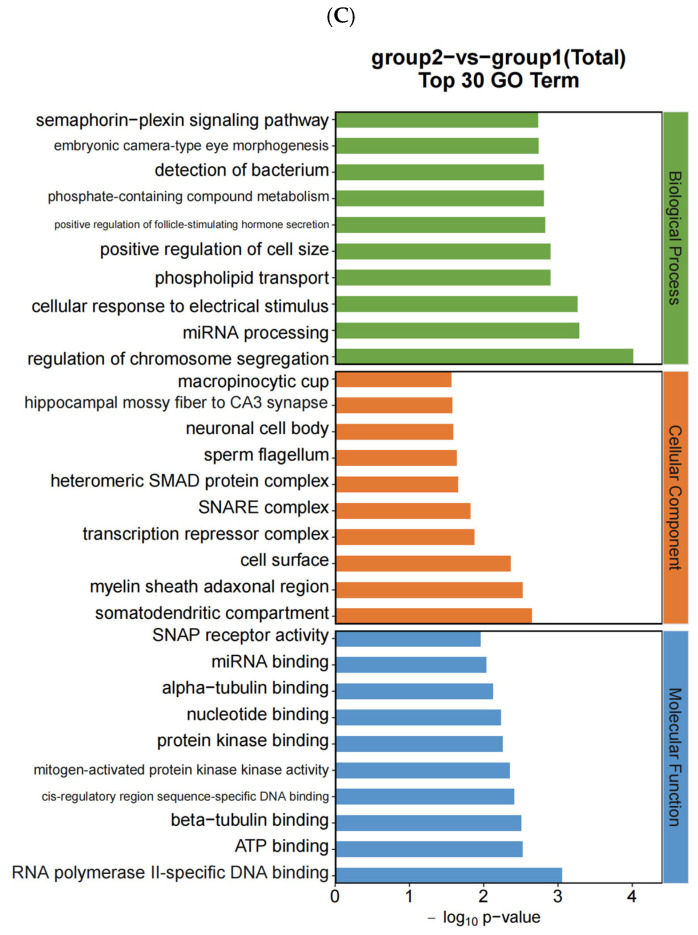

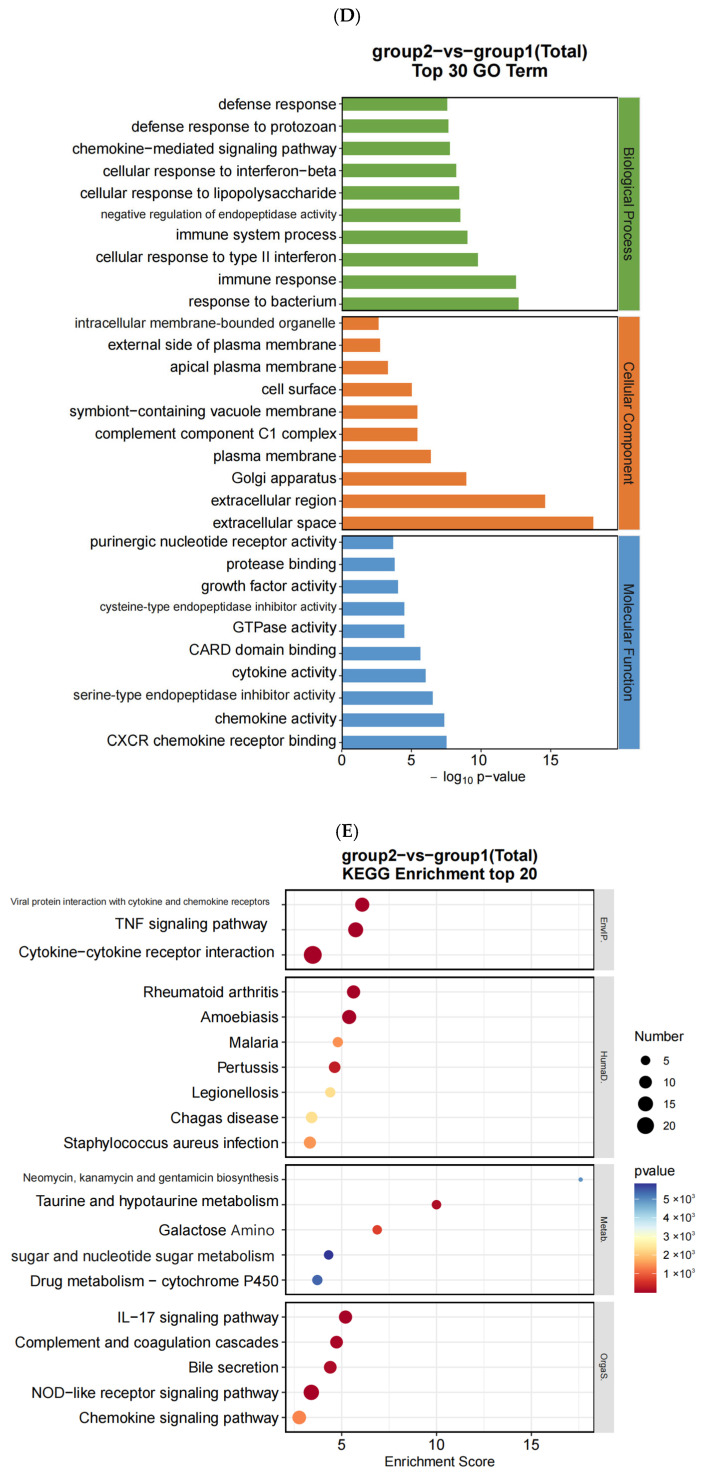

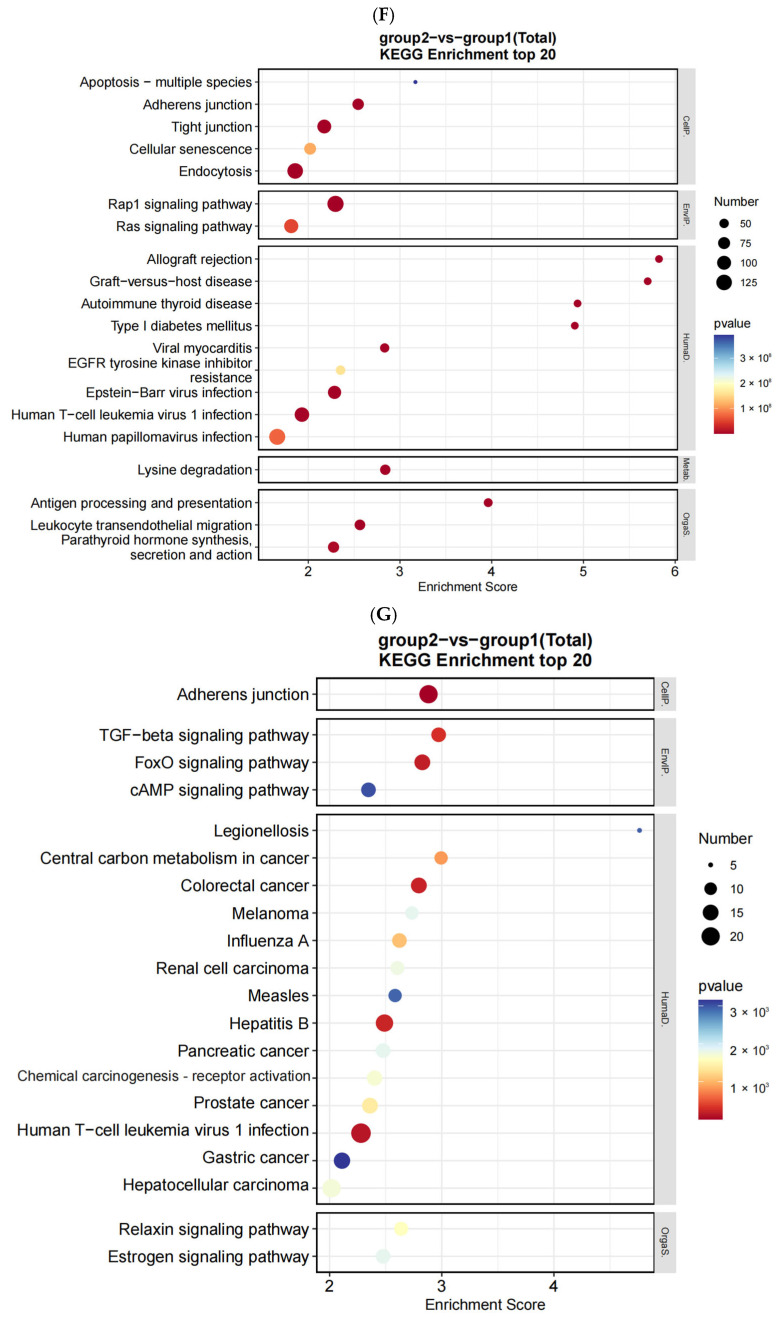

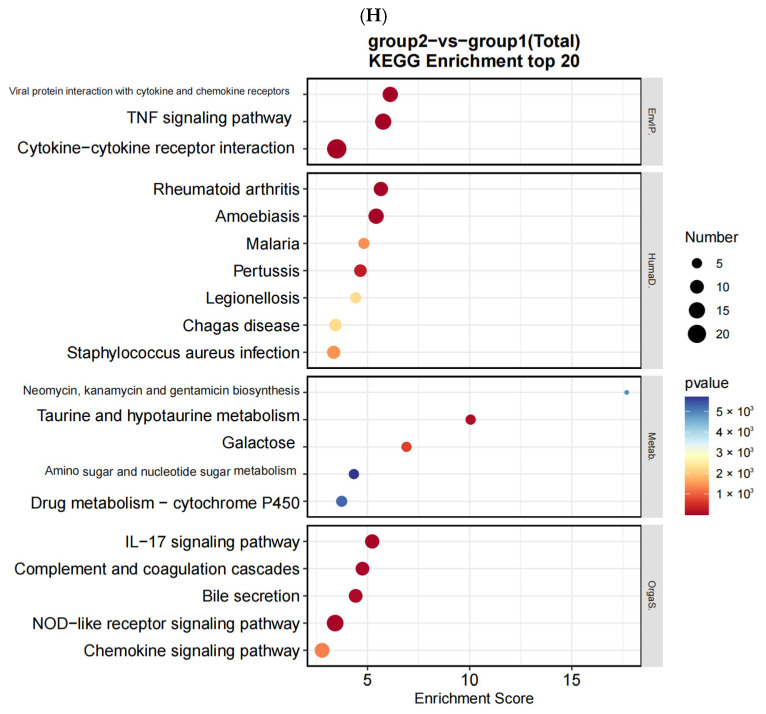
GO enrichment scatter plots and KEGG enrichment scatter plots of differentially expressed mRNA, miRNA, circRNA, and lncRNA in AML12 cells. Comparison results between Group 2 (0.8 μmol/L treatment group) and Group 1 (control group) (*n* = 3). (**A**) GO enrichment of differentially expressed mRNAs. (**B**) GO enrichment of differentially expressed miRNAs. (**C**) GO enrichment of differentially expressed circRNAs. (**D**) GO enrichment of differentially expressed lncRNAs. (**E**) KEGG enrichment of differentially expressed mRNAs. (**F**) KEGG enrichment of differentially expressed miRNAs. (**G**) KEGG enrichment of differentially expressed circRNAs. (**H**) KEGG enrichment of differentially expressed lncRNAs.

mRNA: The GO analysis of differentially expressed mRNAs revealed significant enrichment in biological processes related to oxidative stress response, iron metabolism, and lipid peroxidation (Figure 4A). These processes are crucial in understanding the cellular response to neodymium nitrate exposure.

miRNA: The GO analysis of differentially expressed miRNAs highlighted pathways involved in the regulation of gene expression and cellular signaling (Figure 4B).

circRNA: The GO analysis of differentially expressed circRNAs indicated involvement in RNA processing and regulation of transcription (Figure 4C).

lncRNA: The GO analysis of differentially expressed lncRNAs showed enrichment in pathways related to chromatin modification and transcriptional regulation (Figure 4D).

#### 3.3.2. KEGG Analysis:

mRNA: The KEGG analysis of differentially expressed mRNAs identified significant enrichment in pathways such as ferroptosis, oxidative phosphorylation, and the Nrf2 signaling pathway (Figure 4E). These pathways are central to the induction of liver injury by neodymium nitrate.

miRNA: The KEGG analysis of differentially expressed miRNAs highlighted pathways involved in the regulation of apoptosis and cell cycle progression (Figure 4F).

circRNA: The KEGG analysis of differentially expressed circRNAs indicated involvement in pathways related to RNA processing and splicing (Figure 4G).

lncRNA: The KEGG analysis of differentially expressed lncRNAs showed enrichment in pathways related to chromatin remodeling and epigenetic regulation (Figure 4H).

These mRNA, miRNAs, lncRNAs, and circRNAs are primarily involved in the following biological processes:

Response to Oxidative Stress: This process encompasses the cellular response to oxidative stress, including the regulation of antioxidant enzymes and the repair of oxidative damage.

Regulation of Lipid Metabolism: This process involves the synthesis, breakdown, and transport of lipids, which are critical for maintaining the integrity of cell membranes and overall cellular function.

KEGG focuses on biochemical pathways. We compared DEGs in the KEGG database to determine the signaling pathways that genes may be involved in. Figure 4E–H display the KEGG analysis results for differentially expressed mRNA, miRNAs, lncRNAs, and circRNAs. These mRNA, miRNAs, lncRNAs, and circRNAs are mainly involved in the following pathways:

Iron Metabolism Pathway: This pathway involves the absorption, storage, and utilization of iron, which is essential for normal cellular function and antioxidant defense mechanisms.

Ferroptosis Pathway: This pathway involves iron-dependent lipid peroxidation, a form of cell death associated with oxidative stress and iron metabolism dysregulation.

### 3.4. Construction of ceRNA Network

**Figure 5:**
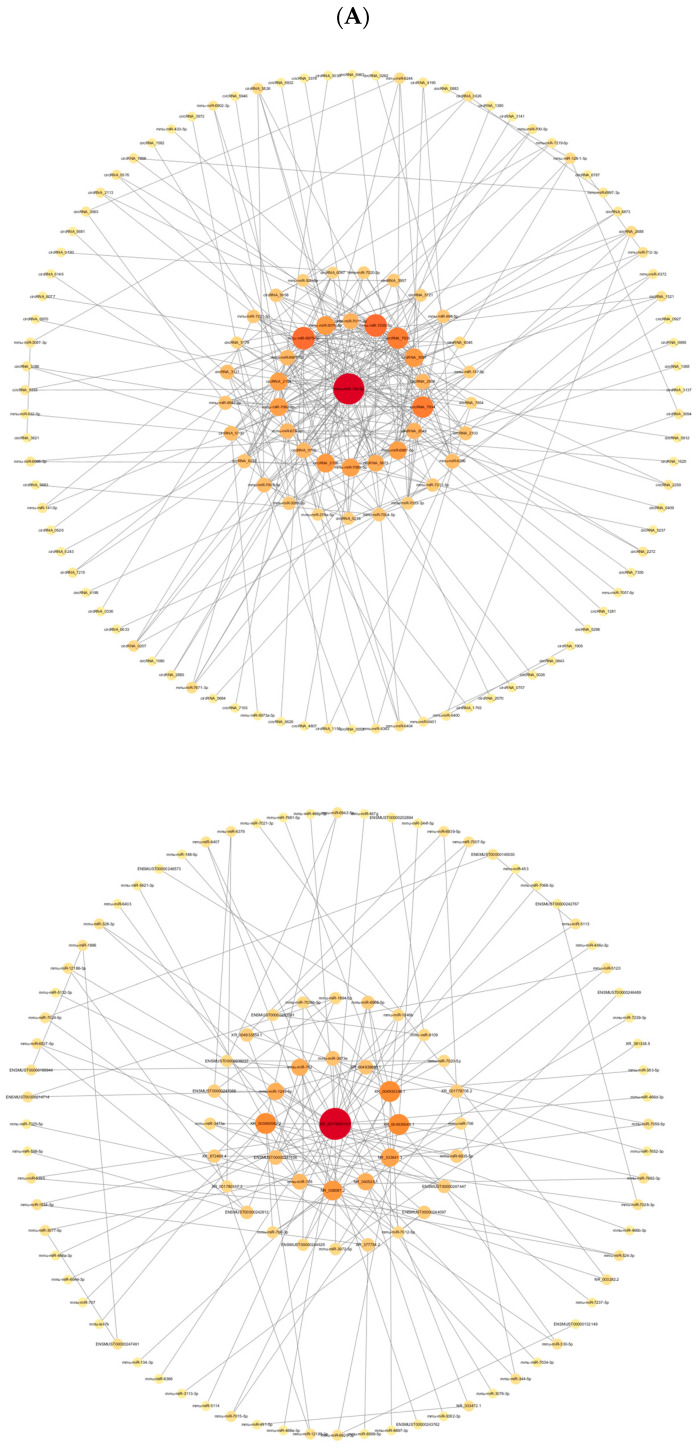

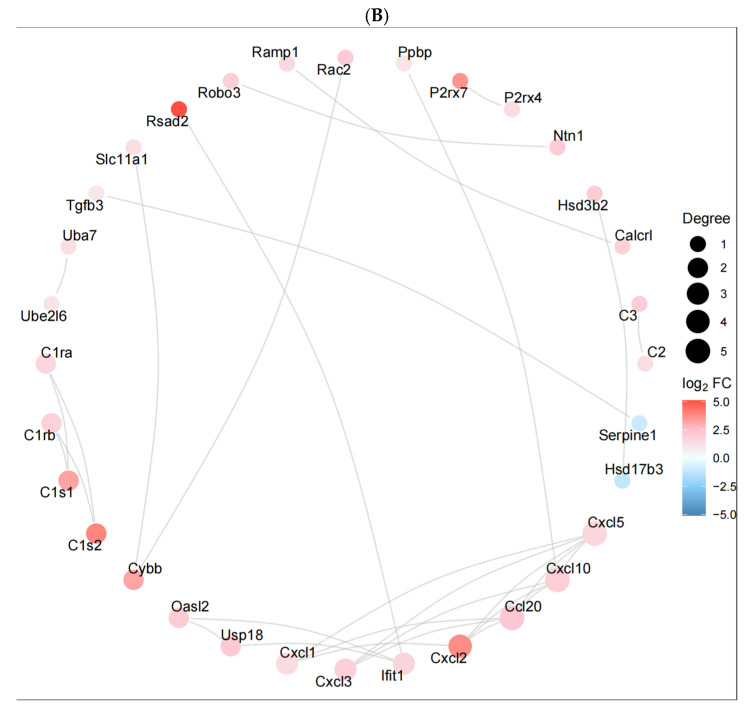
Analysis of ceRNA and PPI for differentially expressed genes. Comparison results between Group 2 (0.8 μmol/L treatment group) and Group 1 (control group) (*n* = 3). (**A**) The ceRNA regulatory network of lncRNA–miRNA–mRNA and circRNA–miRNA–mRNA. Red circles represent lncRNAs or circRNAs, and green triangles represent miRNAs. (**B**) PPI analysis of differentially expressed mRNA, with larger circles indicating more gene interactions, and the top 7 mRNAs highlighted in red.

We predicted the target binding relationships between miRNA and mRNA, lncRNA, and circRNA and then constructed a ceRNA network (Figure 5A) based on common miRNA bindings. The network displays the lncRNA–miRNA–mRNA and circRNA–miRNA–mRNA interactions from the outside in. By constructing the ceRNA network, we identified key regulatory axes that may contribute to the observed changes in gene expression. For instance, specific miRNAs such as miR-149-3p, miR-6975-5p, and miR-7235-5p were found to target multiple genes, indicating their central role in regulating the cellular response to neodymium nitrate exposure.

### 3.5. PPI Analysis of Differential mRNA

Our data analysis of differentially expressed mRNA on the STRING database revealed 333 nodes and 93 edges. The interactome network diagram with Cytoscape’s Betweenness Centrality Analysis (Figure 5B) consists of 33 nodes in total, where larger nodes indicate more mRNA interactions, and red nodes represent the top 7 mRNAs. Complement system C3, chemokine Cxcl10, chemokine Cxcl1, atypical chemokine receptor 3 (Ackr3), bactericidal/permeability-increasing protein (Bpi), monocyte chemoattractant protein-1 (Ccl2), and coagulation factor V (F5) ranked in the top 7 in the PPI network analysis.

### 3.6. The GSEA Enrichment Analysis of Gene Sets in the Reactome Database

**Figure 6:**
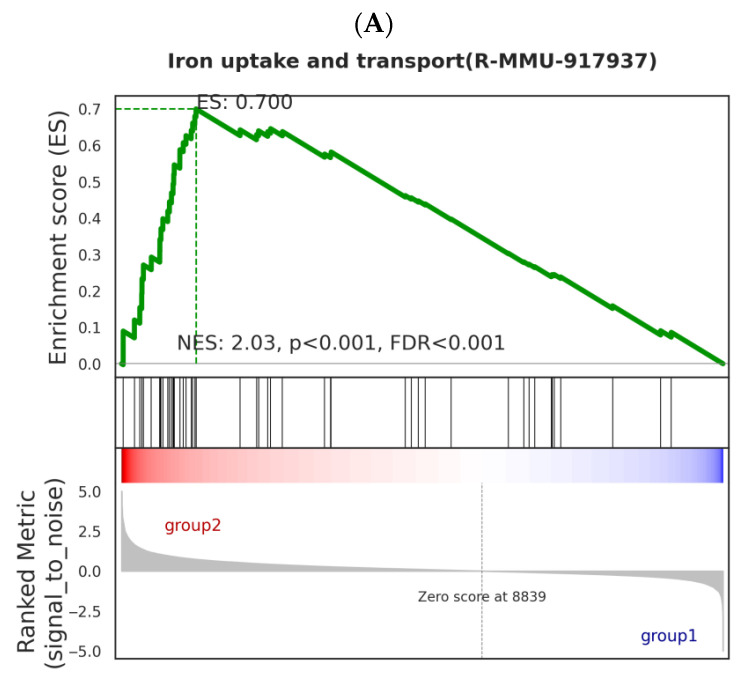

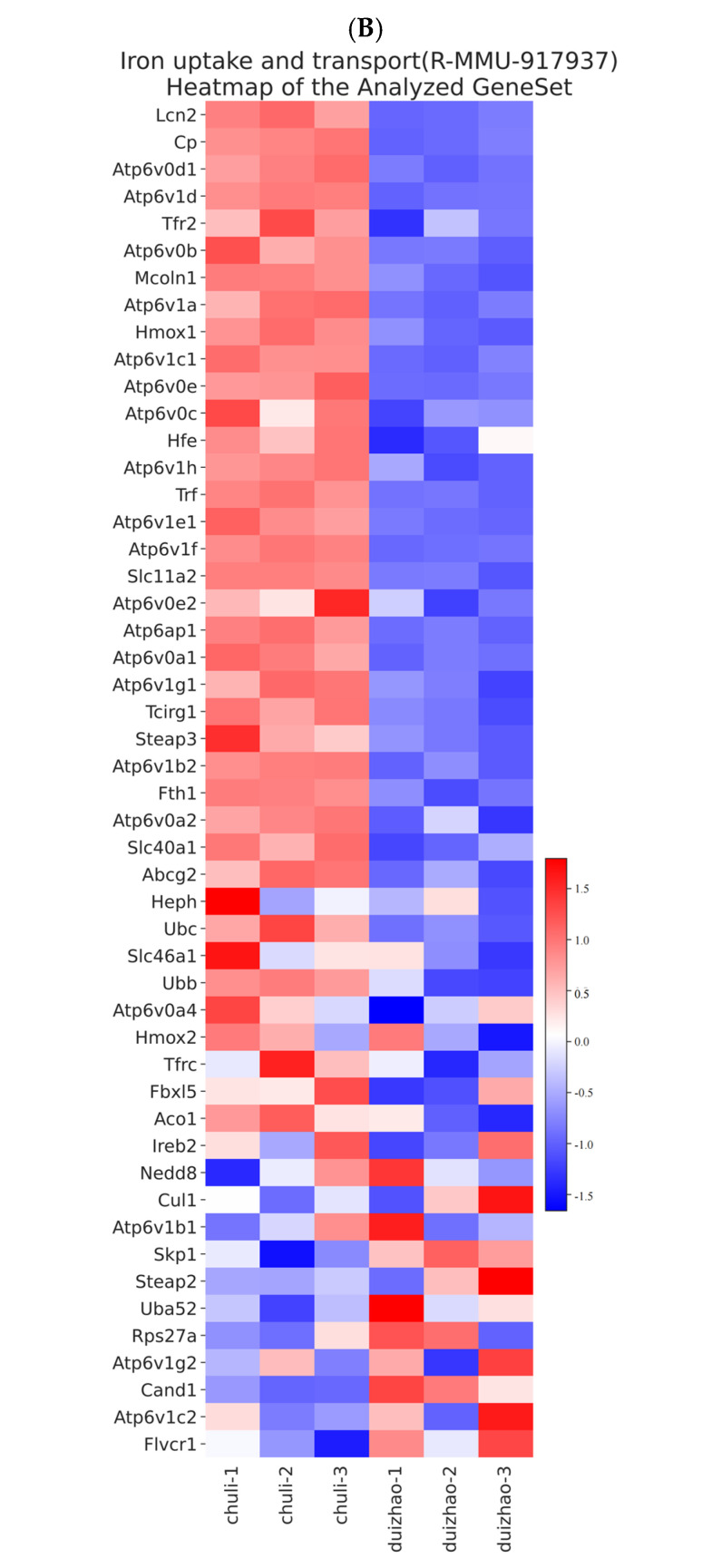
GSEA enrichment analysis of gene sets in the Reactome database. Comparison results between Group 2 (0.8 μmol/L treatment group) and Group 1 (control group) (*n* = 3). (**A**) GSEA enrichment analysis of iron uptake and transport. (**B**) Heatmap analysis of iron uptake and transport.

Our data analysis of enriched mRNA on the Reactome database shows significant enrichment of the iron intake and transport gene set (NES value of 2.03, *p* < 0.01, FDR < 0.001, Figure 6).

### 3.7. Verification of Differential Expression Profiles by qRT-PCR

To confirm the reliability of the RNA sequencing (RNA-seq) results, we validated the expression levels of selected differentially expressed genes (DEGs) using quantitative real-time PCR (qRT-PCR). The genes chosen for validation were Ptgs2, NOX1, Fth1, ACSL4, Gpx4, Slc7a11, and Socs1. These genes were selected based on their biological significance in the context of neodymium-nitrate-induced liver injury and their potential involvement in ferroptosis and oxidative stress pathways.

#### 3.7.1. Rationale for Gene Selection:

Ptgs2 (Prostaglandin-Endoperoxide Synthase 2): Ptgs2 is a key enzyme involved in the synthesis of prostaglandins, which play a crucial role in inflammation and oxidative stress. Elevated levels of Ptgs2 have been associated with liver injury and oxidative stress responses.

NOX1 (NADPH Oxidase 1): NOX1 is a member of the NADPH oxidase family, which generates reactive oxygen species (ROS) and contributes to oxidative stress. NOX1 has been implicated in various forms of liver injury, including those induced by environmental toxins.

Fth1 (Ferritin Heavy Chain 1): Fth1 is a component of ferritin, the major iron storage protein in cells. Changes in Fth1 expression can affect intracellular iron levels and contribute to oxidative stress and ferroptosis.

ACSL4 (Acyl-CoA Synthetase Long-Chain Family Member 4): ACSL4 is involved in lipid metabolism and has been shown to play a role in ferroptosis by promoting lipid peroxidation.

Gpx4 (Glutathione Peroxidase 4): Gpx4 is a crucial antioxidant enzyme that protects cells from oxidative damage by reducing lipid hydroperoxides. Its downregulation is associated with increased susceptibility to ferroptosis.

Slc7a11 (Solute Carrier Family 7 Member 11): Slc7a11 is a key regulator of the cystine-glutamate antiporter system, which is essential for maintaining intracellular glutathione levels and protecting against oxidative stress.

Socs1 (Suppressor of Cytokine Signaling 1): Socs1 is involved in the regulation of cytokine signaling and has been implicated in the modulation of inflammatory responses and oxidative stress.

#### 3.7.2. Validation Results:

We partially validated the differentially expressed mRNAs, miRNAs, and circRNAs. Due to the special circular formation mechanism of circRNAs, we selected circRNAs with reverse splicing reads for validation. The qPCR results were largely consistent with the sequencing data (Figure 6). Compared to the control, the FPKM of the Ptgs2 gene in Nd(NO_3_)_3_ 0.8 µM-treated AML12 cells showed a significant decrease in sequencing (t-value = 8.439, *p*-value = 0.0011), with no significant difference in mRNA expression (t-value = 1.991, *p*-value = 0.1689); the FPKM of the Nox1 gene showed no significant difference in sequencing (t-value = 1.505, *p*-value = 0.2547), with no significant difference in mRNA expression (t-value = 1.506, *p*-value = 0.2627); the FPKM of the Fth1 gene significantly increased (t-value = 13.60, *p*-value = 0.0027), and mRNA expression significantly increased (t-value = 8.353, *p*-value = 0.0016); the FPKM of the ACSL4 gene significantly increased (t-value = 16.12, *p*-value = 0.0006), and mRNA expression significantly increased (t-value = 7.368, *p*-value = 0.0171); the FPKM of the Gpx4 gene significantly increased (t-value = 4.816, *p*-value = 0.0120), and mRNA expression significantly increased (t-value = 4.446, *p*-value = 0.0123); the FPKM of the Slc7a11 gene significantly increased (t-value = 5.761, *p*-value = 0.0152), and mRNA expression significantly increased (t-value = 4.183, *p*-value = 0.0496); the FPKM of the Socs1 gene significantly decreased (t-value = 6.113, *p*-value = 0.0045), and mRNA expression significantly decreased (t-value = 3.187, *p*-value = 0.0490); CircRNA_1156 showed no significant difference in sequencing RBP (t-value = 1.760, *p*-value = 0.2205), but mRNA expression significantly decreased (t-value = 4.768, *p*-value = 0.0100); miR-326-5p showed no significant difference in sequencing TPM (t-value = 2.596, *p*-value = 0.1218), and mRNA expression showed no significant difference (t-value = 1.003, *p*-value = 0.3734).

The qRT-PCR results were largely consistent with the RNA-seq data, confirming the differential expression of these genes in response to neodymium nitrate exposure. For example, the expression of Ptgs2 and NOX1 was significantly upregulated, while Fth1 and Gpx4 were significantly downregulated, supporting their roles in neodymium-nitrate-induced liver injury (Table 2).

By validating these specific genes, we aimed to provide a more comprehensive understanding of the molecular mechanisms underlying neodymium-nitrate-induced liver injury and to identify potential biomarkers and therapeutic targets.

### 3.8. Intracellular ROS and Mitochondrial ROS Measurement

**Figure 7:**
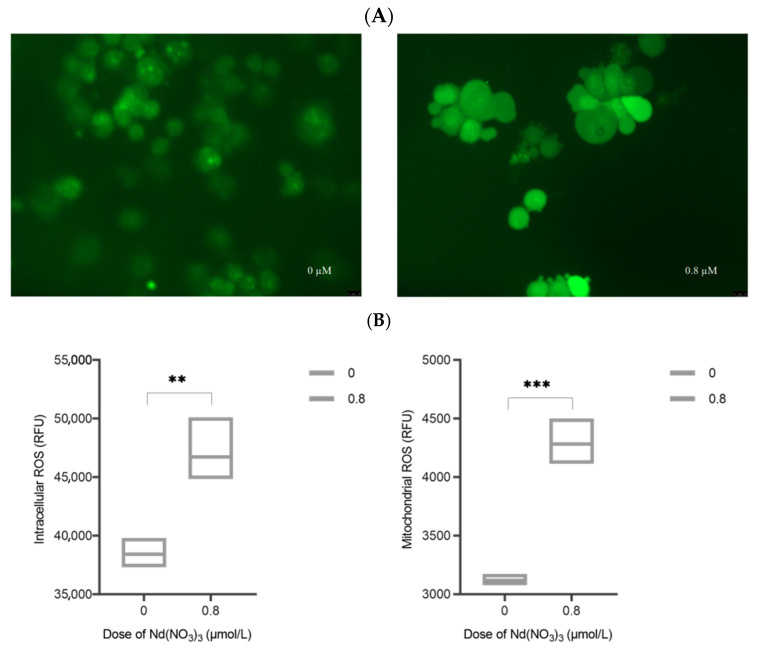

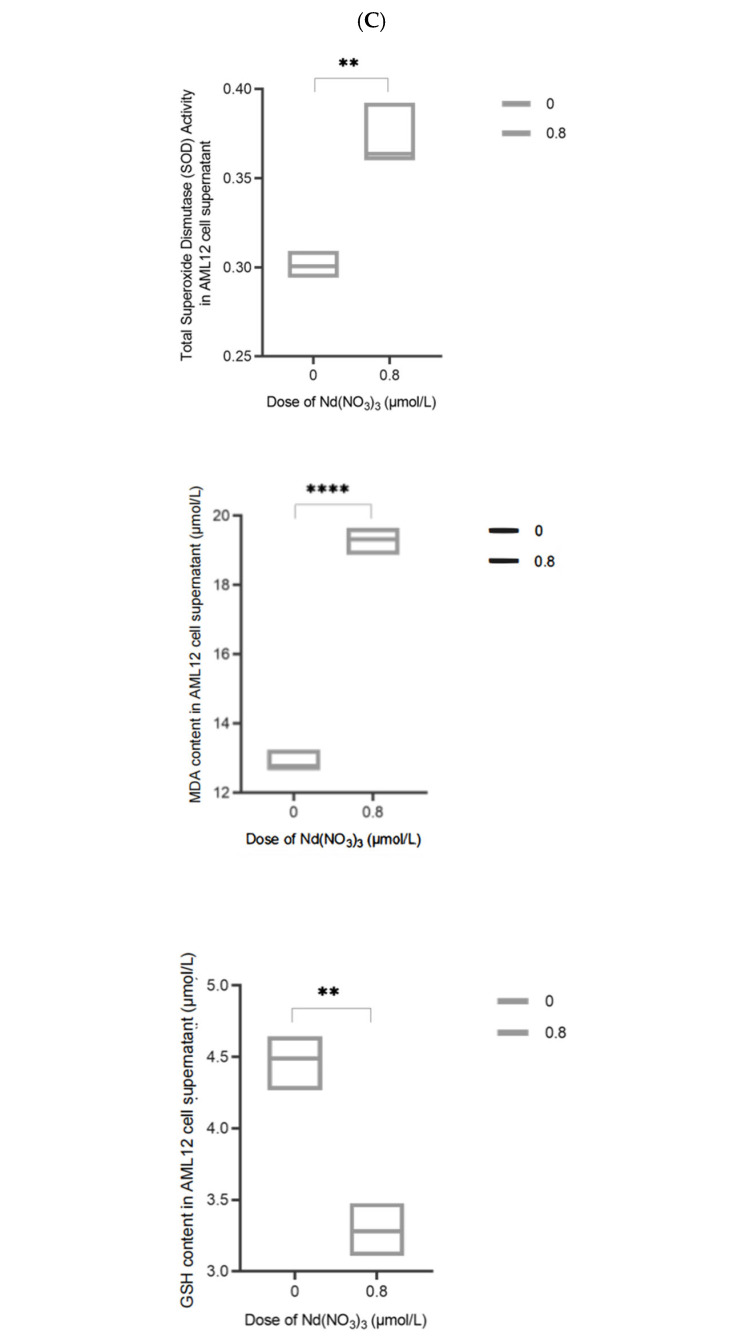
Measurement of intracellular ROS and mitochondrial ROS in AML12 cells after 24 h exposure to 0.8 µM Nd(NO_3_)_3_; determination of ferroptosis-related markers in the cell supernatant (n = 3). (**A**) DCFH-DA assay for intracellular ROS; (**B**) quantitative measurement of intracellular ROS and mitochondrial ROS; (**C**) measurement of superoxide dismutase (SOD) levels, malondialdehyde (MDA) levels, and glutathione (GSH) levels. (**: *p* < 0.01, ***: *p* < 0.001, ****: *p* < 0.0001 compared to the negative control group).

Compared with the control, the green fluorescence intensity of DCFH-DA was more distinct and brighter at 0.8 µM (Figure 7). Compared with the control, intracellular ROS in AML12 cells treated with Nd(NO_3_)_3_ at 0.8 µM were significantly increased (t-value = 5.138, *p*-value = 0.0068), and mitochondrial ROS were also significantly elevated (t-value = 10.21, *p*-value = 0.0005).

It should be noted that the DCFH-DA assay primarily detects hydrogen peroxide (H_2_O_2_) and may not effectively capture all reactive oxygen species (ROS). This limitation should be considered when interpreting the results. Future studies may consider using alternative methods, such as the use of multiple fluorescent probes or electron paramagnetic resonance (EPR) spectroscopy, to provide a more comprehensive assessment of ROS.

### 3.9. Detection of Ferroptosis-Related Markers

Compared with the control, in the supernatant of AML12 cells treated with Nd(NO_3_)_3_ at 0.8 µM, the levels of superoxide dismutase (SOD) were significantly increased (t-value = 6.377, *p*-value = 0.0031), the malondialdehyde (MDA) levels were significantly elevated (t-value = 22.06, *p*-value < 0.0001), and the glutathione (GSH) levels were significantly decreased (t-value = 7.701, *p*-value = 0.0015) (Figure 7).

## 4. Discussion

REEs, which include 17 elements with similar physicochemical properties, have been widely used in industry and modern technology [7]. However, they also pose a range of health and environmental issues. Neodymium and other REEs can enter the human body through respiration, deposit in the alveoli, and cause dysfunction of alveolar epithelial cells, inflammatory responses, fibrosis, and apoptosis [18]. These reactions may be related to the physicochemical properties of REEs, which can affect the local environment in the lungs and trigger a series of pathological changes. After entering the body through the digestive tract and entering the bloodstream, neodymium accumulates mostly in the liver, causing liver damage characterized by hepatocyte necrosis, inflammatory responses, and metabolic disorders [19]. The liver, as the primary detoxification organ, is particularly sensitive to the accumulation of REEs, and its damage may lead to a series of metabolic and immune function impairments [20]. In addition to the liver, REEs may also have toxic effects on other organs such as the heart, kidneys, and brain. These effects may be related to the bioaccumulation of REEs and their ability to interfere with normal cell functions. Neodymium also has certain toxic effects on the reproductive system, which may be related to its impact on oxidative stress and inflammatory responses in the body [21].

In this study, we utilized RNA sequencing to analyze the impact of neodymium nitrate on gene expression in AML12 cells. Our findings revealed significant changes in the expression of ferroptosis-related genes, as well as differentially expressed miRNAs, lncRNAs, and circRNAs. These results suggest that neodymium nitrate may induce hepatocyte injury by modulating iron metabolism and oxidative stress pathways.

In our study, we used a threshold of $\left|log2(Foldchange)\right|$ ≥ 1 and a *p*-value ≤ 0.05 to identify differentially expressed genes (DEGs). This approach allows us to capture genes with moderate expression changes that may still play a significant role in the biological processes underlying neodymium-nitrate-induced liver injury. While a stricter threshold like logFC > 2 could provide a more stringent filter for identifying DEGs, it may exclude genes with moderate expression changes that are biologically relevant. Future studies may consider using a combination of thresholds or additional validation methods to further refine the identification of DEGs.

In conclusion, the choice of threshold depends on the specific research question and the biological context. Our study aims to provide a comprehensive overview of the gene expression changes induced by neodymium nitrate, and we believe that using $\left|log2(Foldchange)\right|$ ≥ 1 strikes a balance between sensitivity and specificity.

In this study, we investigated the effects of neodymium nitrate on gene expression and cellular pathways in AML12 hepatocytes. Our findings reveal significant changes in the expression of genes related to ferroptosis and oxidative stress, indicating that neodymium nitrate induces liver injury through the modulation of iron metabolism and lipid peroxidation pathways.

In the context of liver injury, the accumulation of neodymium in hepatocytes can lead to significant oxidative stress, as evidenced by increased levels of ROS and oxidative damage markers such as 8-hydroxydeoxyguanosine (8-OHdG) and γ-H2AX. These changes can disrupt normal cellular functions and lead to hepatocyte necrosis and inflammation, ultimately contributing to liver damage [17]. Understanding the specific mechanisms by which neodymium induces oxidative stress and activates downstream signaling pathways can provide valuable insights into the development of therapeutic strategies to mitigate its toxic effects. As illustrated in Figure 1, the ferroptosis axis (GPX4↓/MDA↑) constitutes a core mechanism in Nd hepatotoxicity, aligning with pathway enrichment analyses (Section 3.3).

Our study identified significant changes in the expression of non-coding RNAs (ncRNAs) in AML12 cells exposed to neodymium nitrate (Nd(NO_3_)_3_). These findings are consistent with recent studies that have highlighted the role of small non-coding RNAs in mediating the effects of environmental pollutants. For instance, a study by [12] demonstrated that small non-coding RNAs play a crucial role in the response to environmental pollution, influencing gene expression and cellular pathways involved in stress response and detoxification processes [13]. This suggests that ncRNAs may also be involved in the hepatotoxic effects of Nd(NO_3_)_3_, potentially contributing to the observed gene expression changes and liver injury. Understanding the involvement of ncRNAs in Nd(NO_3_)_3_-induced liver injury could provide valuable insights into the underlying mechanisms and potential biomarkers for early detection and intervention. The strand-specific RNA-seq approach (Figure S1) ensured accurate quantification of antisense transcripts, particularly critical for lncRNA analysis.

ceRNA and PPI network analyses provide a perspective on the complex interactions between genes and miRNAs in liver injury induced by Nd(NO_3_)_3_. These networks help us understand the molecular mechanisms regulating liver injury and reveal potential biomarkers [22]. By predicting the target binding relationships between miRNAs and mRNAs, lncRNAs, and circRNAs, ceRNA networks were constructed. This network reveals the complex regulatory relationships between non-coding RNAs and coding RNAs, with miRNAs in a central regulatory position, capable of regulating multiple target genes simultaneously. The ceRNA network shows the interactions between lncRNA–miRNA–mRNA and circRNA–miRNA–mRNA. These interactions may affect the availability of specific miRNAs, thereby influencing the expression of their target mRNAs and participating in the development of liver injury. PPI network analysis shows the physical interactions between proteins encoded by differentially expressed mRNAs, which may play a central role in liver injury. Key nodes in the network (such as the complement system C3, chemokine Cxcl10, Cxcl1, etc.) may represent key regulatory genes and signaling nodes in liver injury induced by Nd(NO_3_)_3_. These chemokines play a key role in inflammation and immune cell attraction and may play an important role in liver injury induced by Nd(NO_3_)_3_. Ackr3, as a non-signaling chemokine receptor, may be involved in regulating the availability of chemokines, affecting inflammatory responses. Bpi, bactericidal/permeability-increasing protein, may play a role in anti-infection and immune regulation. Ccl2, monocyte chemoattractant protein-1, may attract monocytes and other immune cells to the site of injury, exacerbating or regulating inflammatory responses. F5, coagulation factor V, may be involved in blood coagulation and wound healing processes, and its abnormalities may be related to liver injury. For miR-149-3p, miR-6975-5p, and miR-7235-5p, these miRNAs target the most genes in the ceRNA network, indicating that they may play a central role in regulating liver injury. They may regulate multiple biological processes by targeting multiple genes simultaneously [18]. Differentially expressed genes and miRNAs, due to their key roles in liver injury induced by Nd(NO_3_)_3_, may serve as biomarkers for early diagnosis and prognosis assessment. The ceRNA network not only provides a comprehensive view of the regulatory interactions but also serves as a valuable resource for identifying potential biomarkers and therapeutic targets. By pinpointing key nodes in the network, we can identify critical regulatory elements that could be targeted for therapeutic intervention. These findings provide new directions for future research and may help develop new prevention and treatment strategies.

In the GSEA analysis, significant enrichment of pathways such as iron intake and transport was discovered, which has an important relationship with Nd(NO_3_)_3_-induced liver injury. The enrichment of iron intake and transport genes may indicate that Nd(NO_3_)_3_ exposure leads to an imbalance in iron metabolism within hepatocytes. Excessive accumulation of iron can trigger the production of reactive oxygen species (ROS) within cells, leading to oxidative stress. Oxidative stress is one of the important mechanisms of hepatocyte injury and death [23]. Ferroptosis is a form of cell death that depends on iron, characterized by the accumulation of lipid peroxides within cells [24]. The enrichment of iron intake and transport genes in the GSEA analysis suggests that Nd(NO_3_)_3_ may induce ferroptosis by promoting iron intake or inhibiting iron excretion, leading to an excessive accumulation of iron. Our experimental results show that compared with the control group, the ROS level in Nd(NO_3_)_3_-treated AML12 cells increased significantly. This finding is consistent with the pathological characteristics of ferroptosis, which is a form of cell death associated with increased intracellular iron levels and lipid peroxidation. The accumulation of ROS is a key factor in the process of ferroptosis because it can react with polyunsaturated fatty acids in the cell membrane to form lipid peroxides, leading to cell membrane damage and cell death. Nd(NO_3_)_3_ exposure may lead to increased oxidative stress in AML12 cells, which may be due to the disruption of the intracellular redox balance by Nd(NO_3_)_3_. The increase in oxidative stress may be related to the disruption of iron metabolism within cells induced by Nd(NO_3_)_3_ as iron is a key metal ion that catalyzes the generation of reactive oxygen. In the context of ferroptosis, the accumulation of iron can promote the Fenton reaction, thereby producing more hydroxyl radicals, which are highly reactive ROS. One of the characteristics of ferroptosis is the increase in intracellular iron levels, which is consistent with our finding that the ROS level in Nd(NO_3_)_3_-treated AML12 cells increased. This increased ROS level may be due to the disruption of iron metabolism induced by Nd(NO_3_)_3_, leading to the release of iron from storage proteins such as ferritin, increasing the free iron pool available for ROS production. The significant increase in ROS levels poses a challenge to the cell’s antioxidant defense system. We observed a significant increase in SOD activity in Nd(NO_3_)_3_-treated AML12 cells. SOD is the first line of defense for cells against oxidative stress, capable of catalyzing the dismutation of superoxide anions into oxygen and hydrogen peroxide, thereby reducing the level of ROS within cells. The increase in SOD activity may reflect the cell’s adaptive response to oxidative stress induced by Nd(NO_3_)_3_, attempting to mitigate oxidative damage by enhancing SOD activity. MDA is the end product of lipid peroxidation, and its increased level directly reflects the degree of lipid peroxidation of the cell membrane. In our study, the MDA level in Nd(NO_3_)_3_-treated AML12 cells increased significantly, indicating that the cell membrane suffered severe oxidative damage. This damage may be due to the accumulation of ROS induced by Nd(NO_3_)_3_, especially hydroxyl radicals and singlet oxygen, which can attack polyunsaturated fatty acids, triggering a chain reaction of lipid peroxidation. GSH is one of the most important antioxidants within cells, involved in a variety of antioxidant defense pathways, including the direct elimination of ROS and acting as a cofactor for glutathione peroxidase (GPx). We found that the level of GSH in Nd(NO_3_)_3_-treated AML12 cells changed, which may indicate a change in the cell’s antioxidant status. A decrease in GSH levels may weaken the cell’s antioxidant capacity, making the cell more susceptible to oxidative damage, while an increase in GSH levels may reflect the cell’s adaptive upregulation in response to oxidative stress. The changes in SOD, MDA, and GSH are closely related to the pathological characteristics of ferroptosis. Ferroptosis is characterized by iron-dependent lipid peroxidation, and the increased level of MDA directly reflects this process. In addition, for GSH, as a key regulator of ferroptosis, its level changes may affect the susceptibility to ferroptosis. The depletion of GSH may lead to increased susceptibility to ferroptosis, while an increase in GSH may provide additional antioxidant protection. The changes in these biomarkers are significant for understanding Nd(NO_3_)_3_-induced liver injury. The liver is the main metabolic organ and is particularly sensitive to oxidative stress. Oxidative stress induced by Nd(NO_3_)_3_ may lead to hepatocyte dysfunction, which can affect the liver’s detoxification, metabolism, and immune functions. In addition, sustained oxidative damage may lead to hepatocyte death, triggering inflammatory responses and fibrosis, and ultimately may lead to cirrhosis and liver cancer.

To place our findings within the broader context of current research, we have conducted an extensive review of the literature on neodymium-nitrate-induced liver injury. Our study aligns with previous findings that rare earth elements, including neodymium, can induce oxidative stress and ferroptosis in hepatocytes [25,26]. For instance, a study by [27] demonstrated that neodymium exposure significantly increased the levels of reactive oxygen species (ROS) and lipid peroxidation markers in liver cells, leading to cell death [28]. Similarly, [29] reported that neodymium nitrate altered the expression of genes involved in iron metabolism, further supporting our observations [21,30].

Our study provides novel insights into the molecular mechanisms underlying neodymium-nitrate-induced liver injury. By identifying key genes and pathways involved in ferroptosis and oxidative stress, our research contributes to the growing body of literature on rare earth element toxicity. These findings have important implications for understanding the health risks associated with neodymium exposure and for developing strategies to mitigate its toxic effects.

In conclusion, our study demonstrates that neodymium nitrate induces liver injury through the modulation of iron metabolism and oxidative stress pathways. Future research should focus on further elucidating the molecular mechanisms involved and exploring potential therapeutic interventions.

Our RNA sequencing analysis identified numerous differentially expressed genes (DEGs) in response to neodymium nitrate exposure, primarily involved in ferroptosis, oxidative stress, and lipid metabolism pathways. Key genes such as Ptgs2, NOX1, and ACSL4 were upregulated, while Fth1 and Gpx4, involved in iron storage and antioxidant defense, were downregulated. These changes promote iron-dependent lipid peroxidation, leading to ferroptosis.

Our study provides valuable insights into the hepatotoxic effects of neodymium nitrate (Nd(NO_3_)_3_) and the role of non-coding RNAs in mediating these effects. However, limitations include the use of an in vitro model (AML12 cells) and a specific concentration (0.8 µmol/L) and exposure duration (24 h). Future studies should validate these findings in vivo, explore a wider range of doses and exposure times, and investigate epigenetic effects and the reproductive and neurological impacts of neodymium exposure.

## 5. Conclusions

In summary, our study demonstrates that neodymium nitrate induces liver injury through the modulation of iron metabolism and oxidative stress pathways. The identification of key genes and pathways involved in ferroptosis and oxidative stress provides valuable insights into the molecular mechanisms underlying neodymium-nitrate-induced hepatotoxicity. These findings have important implications for understanding the health risks associated with neodymium exposure and for developing strategies to mitigate its toxic effects. Future research should focus on further elucidating the molecular mechanisms involved and exploring potential therapeutic interventions.

## Figures and Tables

**Table 1:** Global rare earth consumption structure and growth drivers for 2024.

Application Sector	Share (%)	Key Products	Growth Driver
Permanent Magnets	42	NdFeB, SmCo	NEVs (49% demand) and humanoid robots (20–40 kt future demand)
Metallurgy/Mechanical	12	RE alloys	High-strength alloys for aerospace
Petrochemicals	9	Fluid cracking catalysts	Refinery capacity expansion in Asia
Glass/Ceramics	8	Polishing powder and pigments	Semiconductor wafer demand (28% CeO_2_ penetration in 12-inch wafers)
Hydrogen Storage	7	LaNi_5_-based alloys	Grid-scale energy storage revival
Luminescent Materials	7	LED phosphors	MiniLED backlight penetration (+85% YoY)

Source: Adapted from Mordor Intelligence (2024) [9] and CNPowder Industry Report [10].

**Table 2:** qRT-PCR validation results.

Gene Symbol	RNA Type	Fold Change (qRT-PCR)	*p*-Value
Ptgs2	mRNA	2.3 ± 0.4	<0.05
NOX1	mRNA	1.8 ± 0.3	<0.05
Fth1	mRNA	0.5 ± 0.2	<0.05
ACSL4	mRNA	3.1 ± 0.5	<0.01
Gpx4	mRNA	0.6 ± 0.1	<0.01
Slc7a11	mRNA	2.5 ± 0.4	<0.05
Socs1	mRNA	0.4 ± 0.1	<0.05
circRNA_1156	circRNA	0.7 ± 0.2	<0.05
miR-326-5p	miRNA	1.2 ± 0.3	<0.05

## Data Availability

The datasets generated and/or analyzed during the current study are available in the OMIX repository, under the accession number OMIX008773. The RNA sequencing data from AML12 cells exposed to neodymium nitrate have been deposited and are accessible via the provided link: https://ngdc.cncb.ac.cn/omix/release/OMIX008773 (accessed on 2 July 2025).

## Supplementary Materials

The following supporting information can be downloaded at https://www.mdpi.com/article/10.3390/toxics13070573/s1. Figure S1: RNA-seq library construction workflow; Table S1: QC Metrics of RNA-Seq Data; Table S2: Primer sequences used for qPCR validation.
