# Supplementary figures and images for: RNA-Seq Analysis of Mouse Hepatocytes AML12 Exposed to Neodymium Nitrate
Source: Toxics. 2025 Jul 7;13(7):573. doi: 10.3390/toxics13070573 (PMC12299013; doi:10.3390/toxics13070573)

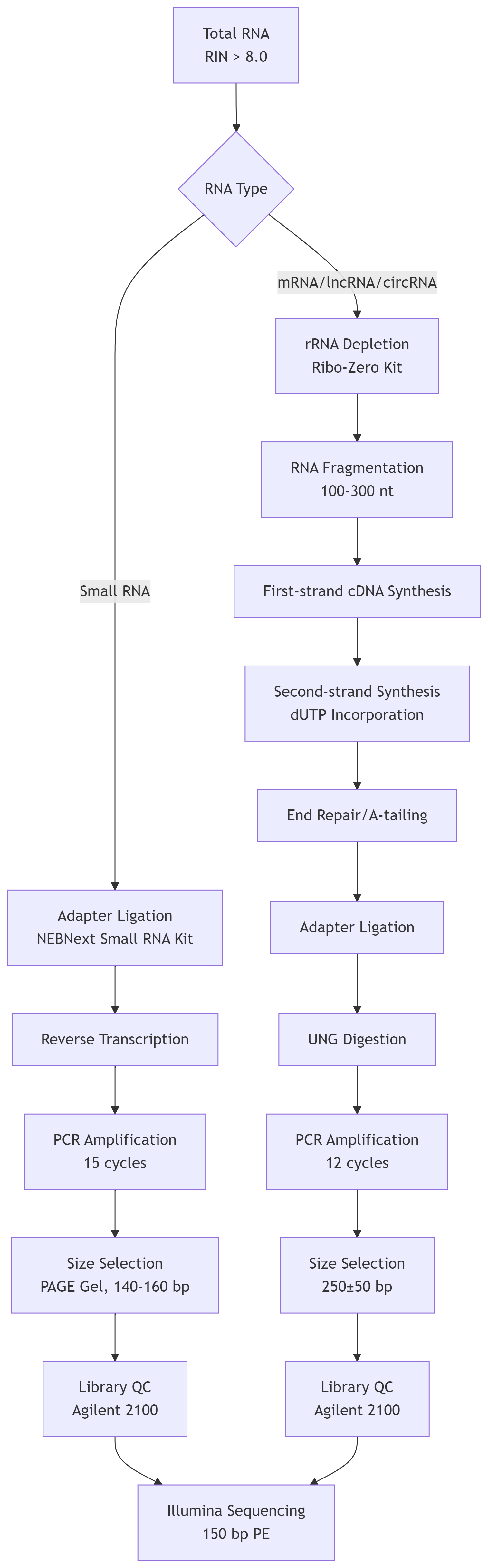

Supplement: Supplementary file 1 [file toxics-13-00573-s001.zip › Supplementary Figure S1. RNA-seq library construction workflow.png]
